# Resveratrol as a cardioprotective adjuvant for 5-fluorouracil in the treatment of gastric cancer cells

**DOI:** 10.1590/1414-431X2024e13537

**Published:** 2024-09-06

**Authors:** Lilong Liu, Yexin Wang, Yanyan Dong, Shan Lin, Wenhui Guan, Jia Song

**Affiliations:** 1 Pharmaceutical Department, The Fourth Affiliated Hospital of Harbin Medical University, Harbin, China; 2 College of Basic Medical Sciences, Harbin Medical University, Harbin, China; 3 Pharmaceutical Department, Qingdao Mental Health Center, Qingdao, China

**Keywords:** 5-Fluorouracil, Cardioctoxicity, Resveratrol, Apoptosis, Oxidative stress, p53

## Abstract

The clinical application of 5-fluorouracil (5-Fu), a potent chemotherapeutic agent, is often hindered by its well-documented cardiotoxic effects. Nevertheless, natural polyphenolic compounds like resveratrol (RES), known for their dual anti-tumor and cardioprotective properties, are potential adjunct therapeutic agents. In this investigation, we examined the combined utilization of RES and 5-Fu for the inhibition of gastric cancer using both *in vitro* and *in vivo* models, as well as their combined impact on cardiac cytotoxicity. Our study revealed that the co-administration of RES and 5-Fu effectively suppressed MFC cell viability, migration, and invasion, while also reducing tumor weight and volume. Mechanistically, the combined treatment prompted p53-mediated apoptosis and autophagy, leading to a considerable anti-tumor effect. Notably, RES mitigated the heightened oxidative stress induced by 5-Fu in cardiomyocytes, suppressed p53 and Bax expression, and elevated Bcl-2 levels. This favorable influence enhanced primary cardiomyocyte viability, decreased apoptosis and autophagy, and mitigated 5-Fu-induced cardiotoxicity. In summary, our findings suggested that RES holds promise as an adjunct therapy to enhance the efficacy of gastric cancer treatment in combination with 5-Fu, while simultaneously mitigating cardiotoxicity.

## Introduction

Gastric cancer (GC) represents a prevalent malignancy of the digestive system, accounting for a considerable proportion of cancer-related deaths worldwide (1). Its pathogenesis is widely recognized as being multistep and multifactorial, characterized by a range of etiologies, genetic changes, and phenotypes. Sadly, the majority of patients are diagnosed with either locally advanced or metastatic disease, underscoring the importance of early detection and intervention. Moreover, the overall survival rate among those diagnosed in advanced stages remains disconcertingly low, hovering between 20-30% (2-4).

Resveratrol (RES; 3, 5, 41-trihydroxystilbene) is a natural polyphenol phytoalexin that is synthesized in various plants such as grapes, peanuts, and herbs (5,6). Numerous preclinical studies have demonstrated RES's multifaceted pharmacological potential, including its anti-cancer, anti-proliferative, anti-inflammatory, and anti-oxidative properties (7,8). RES has also been shown to possess cardio-protective qualities, potentially serving as a chemical prophylactic agent against platelet aggregation while simultaneously preventing and treating atherosclerosis and cardiovascular disease (9,10). Further evidence suggests that the concurrent administration of RES with standard chemotherapy drugs, including 5-fluorouracil (5-Fu), may help up-regulate specific intercellular connections and focal adhesion molecules, inhibit epithelial-stromal transformation, suppress inflammatory pathways, increase cell apoptosis, and enhance chemosensitivity in cancer cells (11-13). Nevertheless, whether RES can mitigate cardiotoxicity induced by 5-Fu remains an open question. Accordingly, the present study aims to explore the synergistic effects of 5-Fu and RES on MFC cells and assess the ability of RES to counteract 5-Fu-induced cardiotoxicity in cardiomyocytes.

## Material and Methods

### Cell cultures

Mouse gastric cancer-derived MFC cell lines were cultured in RPMI-1640 medium (Gibco, USA) supplemented with 10% fetal bovine serum (FBS, Hyclone, USA) and 100 μg/mL penicillin/streptomycin (HaiGene, China) under standard conditions of 37°C with a 5% CO_2_ atmosphere. Resveratrol (purity of ≥99%, Aladdin Chemistry Co. Ltd., China) was dissolved in DMSO as a stock solvent, and used for follow-up studies.

### Primary cultures of rat cardiomyocytes

The hearts of neonatal Sprague-Dawley rats aged 0-3 days were excised and transferred to serum-free DMEM medium (Hyclone), following previously described methods (14-16). The heart tissue was sectioned repeatedly into 1 cubic millimeter blocks, digested with trypsin cell digestion fluid (Beyotime, China), and cardiomyocytes were isolated and cultured in a flask for 2 h to remove fibroblasts. The cells were then seeded at a density of 4×10^8^/L in DMEM medium supplemented with 10% serum and 1% penicillin-streptomycin solution, along with 1% fibrous inhibitor (5-bromo-2-deoxyuridine, BRDU). Finally, the cells were incubated at 37°C under a 5% CO_2_ atmosphere.

### MTT assay

Cells were seeded onto 96-well plates and cultured overnight in an incubator (37°C). Subsequently, cell viability was assessed using 0.5 mg/mL MTT at a temperature of 37°C for a duration of 4 h. Following this, the cells were dissolved with 100 mL of dimethyl sulfoxide (DMSO) and subjected to absorbance measurement at 490 nm.

### Muse annexin V dead cell kit

MFC cells and cardiomyocytes were harvested using distinct protocols. Specifically, MFC cells were cultivated with drugs that were dissolved in a medium containing of 2% serum for a period of 24 h. In contrast, cardiomyocytes were subjected to drug treatment in a serum-free medium following a 12-h period of serum starvation. The cells were categorized into four groups: Control group, 5-Fu group (200 μg/mL), RES group (200 μg/mL), and 5-Fu+RES group (200 μg/L+50 μmol/L). Subsequently, the cells were washed using PBS at a temperature of 4°C and transferred to a tube containing annexin V. Following an incubation period of 20 min at room temperature, cells were analyzed using Muse Cell Analyzer (RWD, China). Finally, the cell mixture was loaded onto the flow cytometer (Agilent, USA) to determine the apoptotic ratio.

### Xenograft models

The MFC cells underwent enzymatic dissociation into individual cells, followed by quantification, and subsequent adjustment of the density to 1×10^6^ cells/mL to generate a tumor cell suspension. Tumor cells were then subcutaneously inoculated into the right dorsum of six-week old BALB/C mice (18-20 g; Liaoning Changsheng Biotechnology Co., Ltd., China), with each mouse receiving an injection volume of 0.2 mL. Following the successful establishment of the gastric cancer model, the experimental animals were randomly allocated to four groups based on their body weights, comprising the tumor model group, the 5-Fu group (20 mg/kg), the RES group (20 mg/kg), and the RES+5-Fu group. 5-Fu was administered intraperitoneally every two days, while RES was administered orally via gavage on a daily basis. The model group received an equivalent volume of saline. Observations encompassed the monitoring of tumor formation and the survival of the mice. Subsequently, after a period of three weeks, the hearts and tumors of the mice were excised for subsequent analyses.

### Cell invasion assay

In accordance with established protocol, the present study utilized 8-μm polyethylene terephthalate filters (BD Pharmingen BioCoatTM Matrigel Invasion Chambers, USA) to conduct a cell invasion assay. To initiate the assay, 200 μL of MFC cells at a concentration of 3×10^5^ cells/mL were allowed to invade through Matrigel-coated filters for a duration of 24 h within a transwell. The invasive capacity of the cells was measured by monitoring the number of cells that successfully migrated to the lower side of the transwell in response to the presence of a chemoattractant comprising 500 μL RPMI-1640 medium supplemented with 10% FBS. Subsequently, cells were fixed using absolute anol and stained using 0.05% crystal violet. A thorough assessment of cell number was performed under a microscope at ×20 magnification (DMi8, Leica, Germany) in 6 randomly chosen fields to accurately quantify the extent of cell invasion.

### Cell migration assay

In keeping with the experimental design, a cell migration assay was conducted, which closely mirrored the previously described cell invasion assay, with the exception that the membrane was not coated with Matrigel. The assay started with 200 μL of MFC cells at a concentration of 5×10^5^ cells/mL being seeded into the upper chambers and allowed to migrate through an 8-μm pore membrane for the designated period. A chemoattractant comprising 500 μL RPMI-1640 medium supplemented with 10% FBS was added to the lower chambers to stimulate cell migration. Following completion of the assay, cells were fixed using absolute anol and stained using 0.05% crystal violet. Quantification of the number of migrated cells was performed under a microscope (DMi8) at ×20 magnification in 6 randomly selected fields to accurately evaluate the extent of cell migration.

### Western blot analysis

The experiment involved lysis and homogenization of drug-treated cells to extract total protein from the supernatant. The protein levels were quantified using the BCA protein assay kit (Beyotime, China). Subsequently, proteins were separated by SDS-PAGE and transferred onto nitrocellulose membranes. To block non-specific binding sites, the membranes were incubated with skim milk powder dissolved in PBS for 90 min at room temperature. Primary antibodies including P53 polyclonal antibodies, Bax polyclonal antibodies (Cell Signaling Technology, USA), LC3 polyclonal antibodies (Elabscience, China), and Bcl-2 polyclonal antibodies (Novus, USA) were added to the membranes at a concentration range of 1:500 to 1:2000 dilution and left overnight. After washing, the membranes were incubated with secondary antibodies for 1 h. Finally, visualization was performed under Odyssey (LI-COR, USA).

### H&E staining

Cardiac tissues of each group of animals were placed in physiological saline at 4°C, fixed in 10% formalin for 48 h, and subsequently subjected to routine paraffin embedding. The tissue samples then underwent the following procedures: gradual deparaffinization with xylene and alcohol, including 15 min in 100% xylene, 10 min in 100% xylene, 10 min in 100% xylene, 10 min in 100% ethanol, 10 min in 95% ethanol, 5 min in 90% ethanol, 5 min in 85% ethanol, and 5 min in 80% ethanol. This was followed by two washes in deionized water, each for 5 min, nuclear staining with hematoxylin for 15 s, a 15-min wash in deionized water, cytoplasmic staining with eosin for 30 s, followed by termination of the coloration with deionized water. Finally, the samples underwent dehydration with a gradient of alcohol, including 30 s in 75% ethanol, 30 s in 80% ethanol, 30 s in 85% ethanol, 30 s in 85% ethanol, 2 min in 90% ethanol, 2 min in 95% ethanol, 5 min in 100% ethanol, 5 min in 100% ethanol, 10 min in 100% xylene, and 10 min in 100% xylene. Subsequently, the samples were sealed in neutral gum, air-dried, and observed under an optical microscope (OLYMPUS, Japan) for pathological evaluation.

### Statistical analyses

Data are reported as means±SE. Statistical analyses including an unpaired two-tailed Student's *t*-test and one-way ANOVA followed by the Bonferroni's multiple comparison *post hoc* test were carried out by using Graphpad Prism v6.0 (USA). Values of P<0.05 were considered significant.

## Results

### RES and 5-Fu synergistically induced MFC cell growth and metastasis arrest

**Figure 1:**
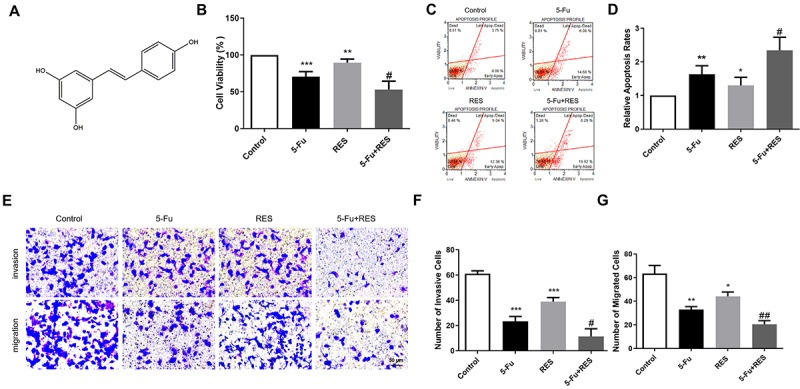
Resveratrol (RES) and 5-fluorouracil (5-Fu) synergistically induced MFC cell growth arrest and cell death. **A**, Chemical structure of resveratrol. **B**, The impact of various treatments, including 5-Fu, RES, and 5-Fu + RES, on the cell viability of MFC gastric cancer cells was evaluated after a 24-h exposure period. **C**, Apoptotic percentage of each group. Graphs were partitioned into four quadrants representing late apoptotic cells, dead cells, live cells, and early apoptotic cells. The apoptotic percentage of the Control group was 10.9±0.5%, 5-Fu group was 17.9±3%, RES group was 14.2±3.5%, and 5-Fu + RES group was 25.8±6.5%. **D**, Histogram depicting the percentage of apoptotic MFC cells. **E**, Transwell assays were used to detect invasive and migratory abilities of MFC cells in the absence or presence of 5-Fu and RES, alone or in combination for 24 h (scale bar 50 μm). **F** and **G**, Histogram of the number of invasive cells and migrated cells. Data are reported as means±SE. *P<0.05, **P<0.01, ***P<0.001 *vs* Control group; ^#^P<0.05 *vs* 5-Fu group; ^##^P<0.01 *vs* RES (n=4, ANOVA).

The structural formula of RES is depicted in Figure 1A. Cell viability analysis revealed a significant decrease in viability upon treatment with both drugs compared to the control group. Moreover, co-administration of RES and 5-Fu led to a significant decrease in cell viability (53.1±11.4%) compared to treatment with either drug alone (Figure 1B). To gain further insights into the underlying mechanism, we used the Muse cell analyzer to determine the apoptotic rate of gastric cancer cells after exposure to 5-Fu and RES. Consistent with the MTT assay results, our findings demonstrated a substantial increase in apoptotic rates in gastric cancer cells following the drug combination treatment (Figure 1C and D). Collectively, our findings suggested that both 5-Fu and RES can stimulate tumor cell apoptosis and exhibit a synergistic effect on gastric cancer cells.

Metastasis, a pivotal step in tumor progression characterized by the migratory and invasive abilities of cancer cells, exerts a significant impact on patient survival. To investigate this phenomenon under controlled conditions, we conducted transwell experiments to evaluate the influence of 5-Fu and RES on the migration and invasion potentials of gastric cancer cells. Our experimental findings demonstrated a significant reduction in the number of stained cells in the lower chamber upon treatment with either 5-Fu or RES alone compared to the control group. Importantly, the combination of these two drugs exhibited a notably more pronounced decrease in the number of stained cells than did each drug alone (Figure 1E top and F). These remarkable observations strongly suggested that both 5-Fu and RES possess the capability to impede cancer cell migration at the cellular level, thereby synergistically exerting their effects. Intriguingly, the outcomes of our cell invasion assay, which employed chambers encapsulated with matrigel, corroborated the findings from the cell migration assay. Specifically, we observed a substantial reduction in cancer cell invasion subsequent to treatment with the combination of 5-Fu and RES, surpassing the reduction achieved by the individual drugs alone (Figure 1E bottom and G). Collectively, these compelling results unequivocally demonstrated the ability of both 5-Fu and RES to effectively inhibit cell migration and invasion. Moreover, when used in combination, they exhibited a synergistic effect, thus highlighting their potential therapeutic significance in mitigating metastasis.

### RES synergistically induced apoptosis with 5-Fu

**Figure 2:**
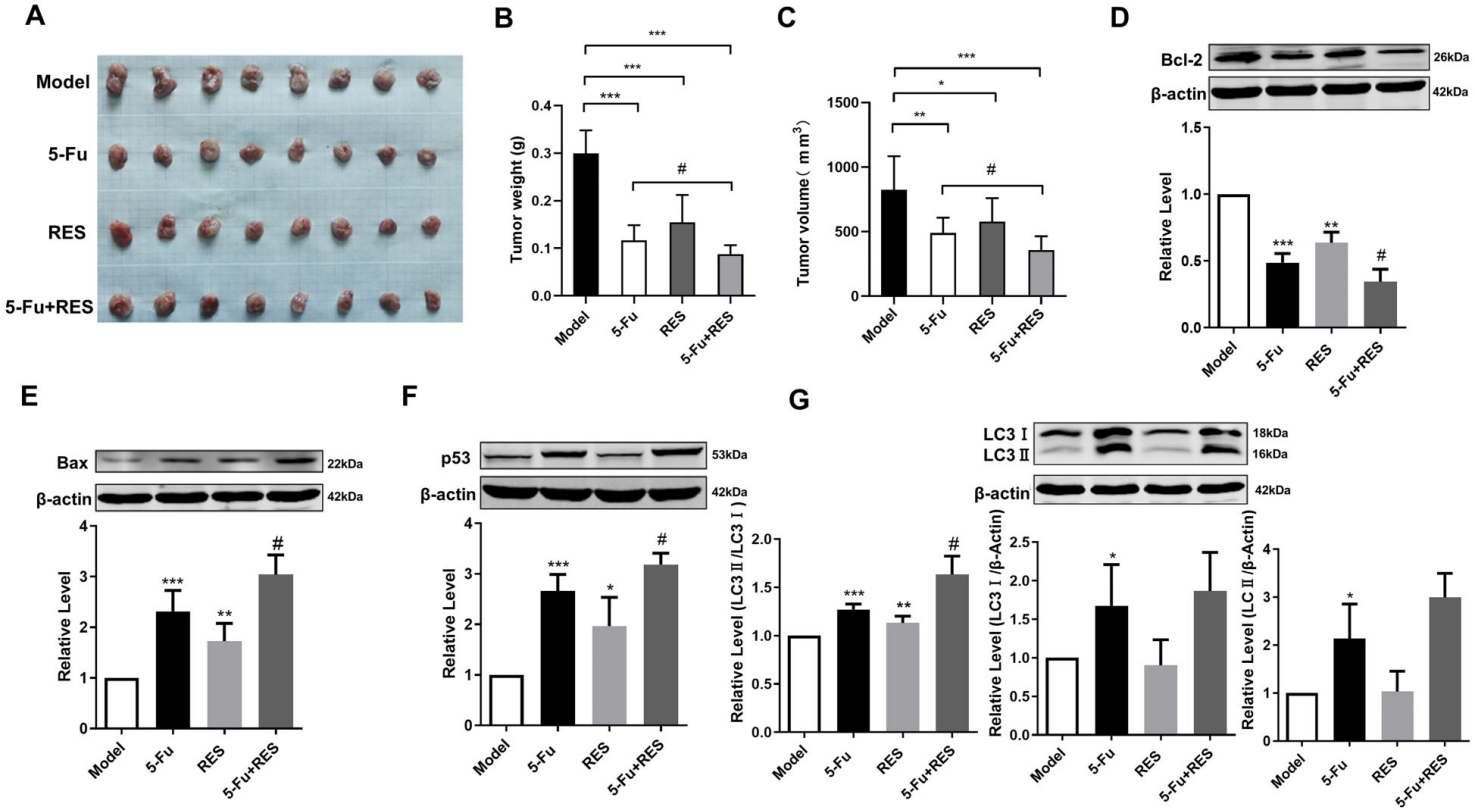
Resveratrol (RES) synergistically with 5-fluorouracil (5-Fu) induced apoptosis. **A**, Representative photograph of tumors from each group. **B**, Statistical analysis of tumor weight. **C**, Statistical analysis of tumor volume (n=8). Effect of RES and 5-Fu on Bcl-2 protein expression (**D**), Bax protein expression (**E**), p53 protein expression (**F**), and LC3 protein expression (**G**) in tumor tissues. Data are reported as means±SE. *P<0.05, **P<0.01, ***P<0.001 *vs* Control (Model) group; ^#^P<0.05 *vs* 5-Fu group (n=3, ANOVA).

To assess the potential inhibitory effects of a combination therapy involving RES and 5-Fu on the progression of GC, we established a xenograft tumor model of gastric cancer. At the termination point of the experiment, tumor tissue analysis demonstrated a significant reduction in both tumor mass and volume in the group receiving the combination therapy of RES and 5-Fu compared to the group treated with 5-Fu alone (Figure 2 A-C).

To elucidate the underlying mechanisms through which the combination of RES and 5-Fu inhibited the progression of GC, we postulated that their synergistic effect might be attributed to an enhanced induction of apoptosis. Based on the results obtained from the Muse analysis, we proposed that the combination treatment of RES and 5-Fu might induce a higher degree of apoptosis. To test this hypothesis, we assessed the levels of apoptotic proteins Bax and Bcl-2 in the mitochondrial pathway. Bcl-2 inhibits apoptosis by preventing the release of apoptosis-inducing factors, such as cytochrome C, from mitochondria. Conversely, Bax facilitates apoptosis by binding to voltage-dependent ion channels on mitochondria, thereby promoting the release of cytochrome C (17). Our findings revealed that the combination treatment of RES and 5-Fu significantly suppressed the expression of Bcl-2 while concurrently elevating the level of Bax to a greater extent compared to treatment with either agent alone (Figure 2D and E).

The p53 protein, a well-known tumor suppressor, plays a pivotal role in regulating the process of apoptosis by interacting with key proteins like Bax and Bcl-2. Through upregulating Bax expression and downregulating Bcl-2 expression, p53 actively promotes apoptosis (18). Our results exhibited a significant increase in p53 expression levels upon treatment with both 5-Fu and RES compared to the control group. Furthermore, co-administration of 5-Fu and RES led to a more substantial upregulation of p53 expression compared to treatment with 5-Fu alone (Figure 2F). Together, the results demonstrated that the combination of RES and 5-Fu induced apoptosis in GC.

In addition to its role in apoptosis regulation, p53 is also implicated in the control of autophagy. Within the nucleus, p53 exerts its transcription-dependent functions and can induce autophagy by inhibiting the mTOR pathway. Autophagy plays a crucial role in inhibiting the accumulation of reactive oxygen species (ROS) in tumor cells and eliminating metabolic waste products, thereby influencing tumor growth, metastasis, drug resistance, and stemness. Numerous small molecules that promote cellular autophagy have been identified and employed in cancer therapy (19). One widely used marker for assessing autophagy is the microtubule-associated protein light chain 3 (LC3) (20). We employed western blot analysis to investigate the effects of 5-Fu and RES on autophagy-related markers. Our results indicated that 5-Fu treatment significantly upregulated the expression levels of LC3-I and LC3-II, consistent with the induction of autophagy in tumor cells. Notably, the combination treatment of 5-Fu and RES did not affect the expression of LC3-I and LC3-II but led to an increase in the LC3-II/LC3-I ratio (Figure 2G). Based on our findings, it can be suggested that the combined treatment with 5-Fu and RES promoted autophagy in tumor cells. Collectively, our study demonstrated that the combination of RES and 5-Fu had the ability to modulate p53-mediated apoptosis and autophagy, thereby exerting inhibitory effects on the proliferation, migration, and invasion of MFC cells.

### RES enhanced cardioprotection against 5-Fu-induced myocardial injury

**Figure 3:**
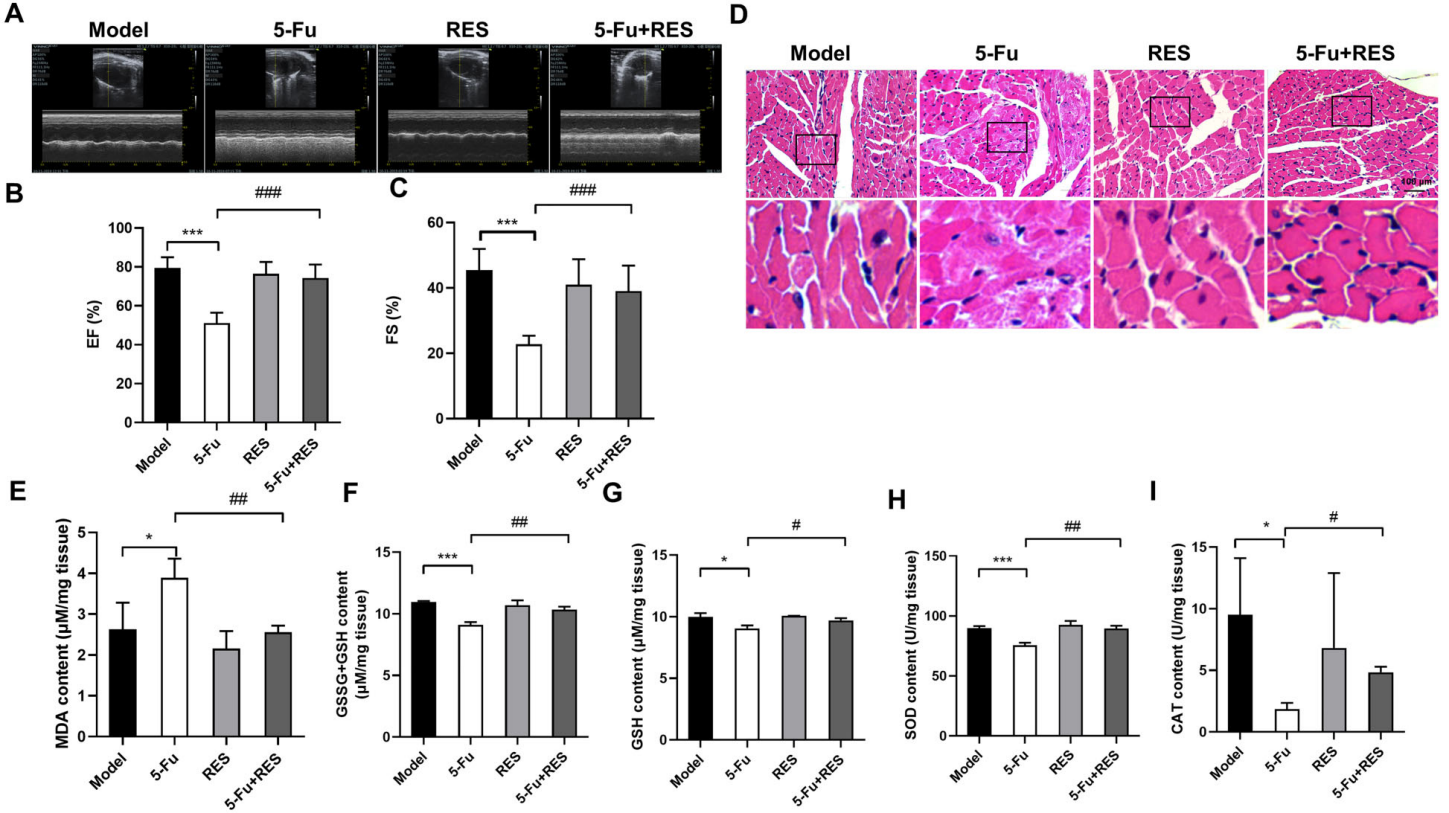
Resveratrol (RES) enhanced cardioprotection against 5-fluorouracil (5-Fu)-induced myocardial injury. **A**, Representative cardiac ultrasound image of each group. **B**, Statistical analysis of cardiac ejection fraction (EF) in each group. **C**, Statistical analysis of the short-axis shortening (FS) rate of the left ventricle in each group. **D**, Representative images of HE staining of myocardial tissue (scale bar 100 μm). **E**, Malondialdehyde (MDA) levels, **F**, total glutathione (GSSG+GSH) levels, **G,** reduced GSH levels, **H**, superoxide dismutase (SOD) levels, and **I**, catalase (CAT) levels in mouse heart tissues of each group. Data are reported as means±SE. *P<0.05, ***P<0.01 *vs* Control (Model) group; ^#^P<0.05, ^##^P<0.01, ^###^P<0.001 *vs* 5-Fu group (n=3, ANOVA).

Cardiac function assessment was performed using small animal sonography to evaluate the left ventricular ejection fraction (EF) and left ventricular short-axis shortening (FS) of the murine heart. As depicted in Figure 3A-C, the EF (79.42±2.26%) and FS (45.40±2.65%) values of the control group were compared with those of the test subjects. A marked reduction in both EF (51.16±2.15%) and FS (22.78±1.06%) was observed in the 5-Fu group (P<0.001), indicating severe cardiac impairment. Notably, the combined treatment group displayed significantly higher values of EF (74.26±2.82%) and FS (39.05±3.18%) than the 5-Fu group (P<0.001), thereby suggesting an improvement in cardiac function. Furthermore, histological examination using H&E staining revealed that myocardial cells in both the model and RES groups displayed regular alignment with stable structures and no apparent pathological alterations. Conversely, the 5-Fu group exhibited focal tissue with unclear boundaries, disordered myocardial cell arrangement, myocardial cell fracture lysis, necrosis of myocardial fibers, lymphocytic infiltration, and severe myocardial injury. In contrast, the extent of myocardial injury was significantly reduced in the RES and 5-Fu combination treatment group (Figure 3D). Taken together, these data suggested that resveratrol administration can ameliorate the decline in cardiac function induced by 5-Fu treatment.

### RES improved oxidative stress in myocardial tissue induced by 5-Fu

Existing research has provided evidence linking the cardiotoxic consequences of 5-Fu to the initiation of intracellular oxidative stress, followed by apoptosis in cardiomyocytes. The escalation of oxidative stress levels stimulates the production of ROS and malondialdehyde (MDA) within the cardiac tissue, thereby causing a decrease in the levels of antioxidant molecules such as total glutathione (oxidized glutathione + reduced glutathione, GSSG + GSH), reduced GSH, and a reduction in the activity of cardiac antioxidant enzymes, including catalase (CAT) and superoxide dismutase (SOD) (21). Consequently, we investigated the impact of RES on oxidative stress induced by 5-Fu administration. Our experimental analysis demonstrated that the administration of 5-Fu substantially elevated the concentration of MDA and decreased the levels of total GSH, reduced GSH, CAT, and SOD within the myocardial tissue. Remarkably, the concurrent utilization of RES and 5-Fu exhibited a significant amelioration in these indicators, indicating a notable mitigation of cardiac oxidative stress (Figure 3E-I).

**Figure 4:**
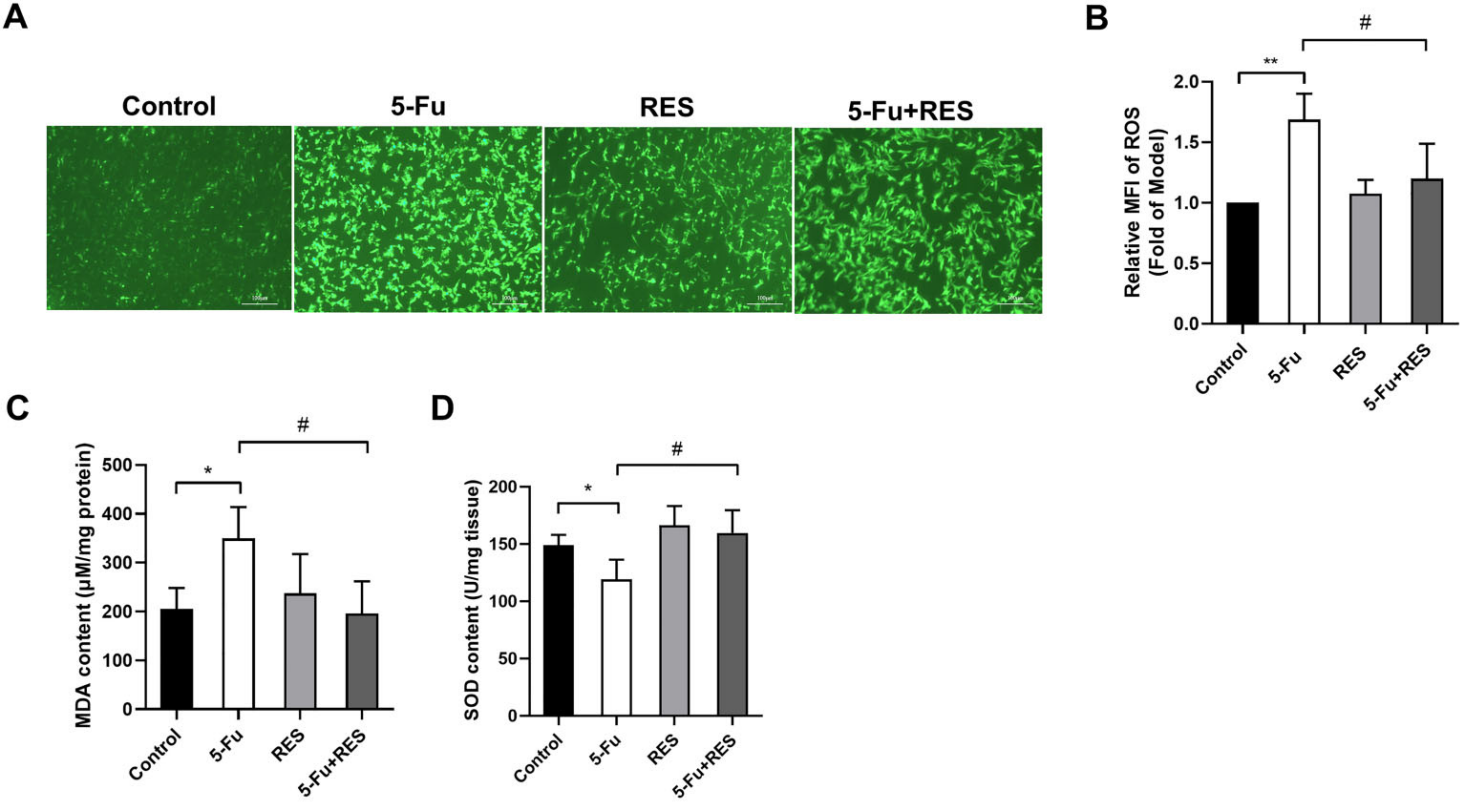
Resveratrol (RES) alleviated 5-fluorouracil (5-Fu)-induced oxidative stress in cardiomyocytes. **A**, Green fluorescence intensity represents the level of reactive oxygen species (ROS) in myocardial cells (scale bar 100 μm). **B**, Statistical graph of fluorescence intensity of cardiomyocytes in each group. **C** and **D**, Statistical graphs of changes in malondialdehyde (MDA) levels and superoxide dismutase (SOD) levels in cardiomyocytes of each group. Data are reported as means±SE. *P<0.05, **P<0.01 *vs* Control group; ^#^P<0.05 *vs* 5-Fu group (n=3, ANOVA).

In order to delve deeper into the impact of RES on 5-Fu-induced oxidative stress, we employed an experimental approach involving the isolation of primary rat cardiomyocytes. Subsequently, these cells were subjected to individual or combined treatments of 5-Fu and RES. Notably, cardiomyocytes stimulated with 5-Fu exhibited a significant accumulation of ROS, as indicated by a substantial increase in the intensity of green fluorescence. This phenomenon was effectively reversed upon the administration of RES. Importantly, similar to the outcomes observed in cardiac tissue, RES supplementation yielded improvement in oxidative stress-related markers within the cardiomyocytes. Collectively, these findings supported that RES possesses the ability to attenuate oxidative stress levels induced by 5-Fu both *in vivo* and *in vitro*, consequently providing myocardial protection (Figure 4A-D).

### RES inhibited apoptosis and autophagy in cardiomyocytes promoted by 5-Fu

**Figure 5:**
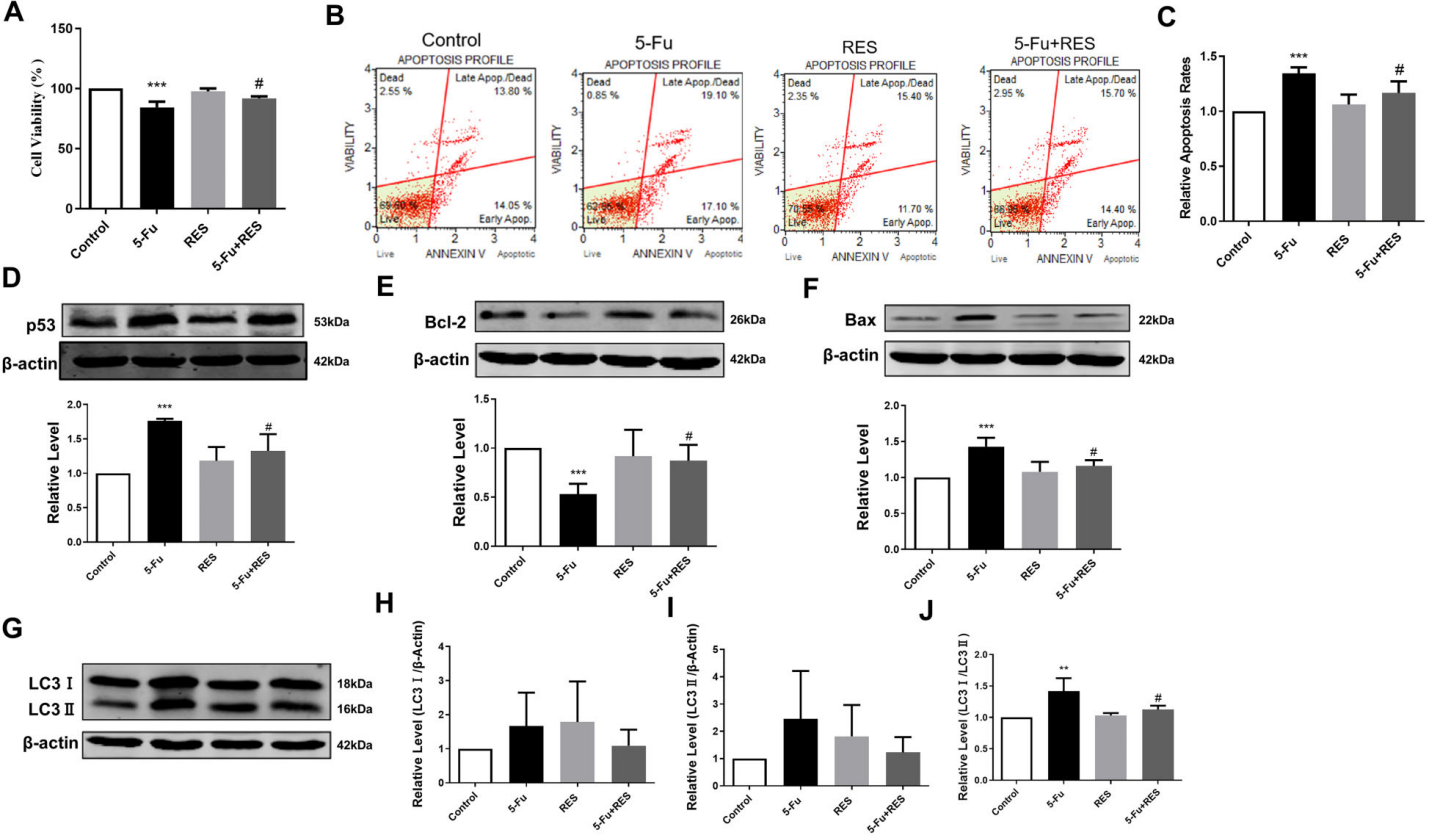
Resveratrol (RES) inhibited apoptosis and autophagy in cardiomyocytes promoted by 5-fluorouracil (5-Fu). **A**, Cell viability of MFC gastric cancer cells after 24 h treatment in control, 5-Fu, RES, and 5-Fu + RES groups. **B**, Apoptotic percentage of primary cardiomyocyte. The apoptotic percentage of 5-Fu group was 32.1±3.9%, RES group was 25.2±2.4%, and 5-Fu+RES group was 27.8±3.5%. **C**, Histogram of the relative apoptotic rate of MFC cells. **D**-**I**, Expression of p53 protein, Bcl-2 protein, Bax protein, and LC3-I and LC3-II proteins in primary cardiomyocytes. **J**, Protein ratio of LC3-I/β-actin and LC3-II/β-actin. Data are reported as means±SE. **P<0.01, ***P<0.001 *vs* Control group; ^#^P<0.05 *vs* 5-Fu group (n=4, ANOVA).

Cardiomyocyte apoptosis, which serves as a marker of cardiac damage caused by 5-Fu, was investigated in response to RES treatment. To assess the effect of RES on cardiomyocyte apoptosis, MTT experiments were conducted to evaluate cell viability. It was observed that 5-Fu led to a significant decrease in cell viability, while RES alone exhibited no notable impact on cardiomyocytes. Interestingly, co-administration of RES and 5-Fu resulted in increased cell viability compared to the group treated with 5-Fu alone (Figure 5A). Further investigation into the influence of these compounds on cardiomyocyte apoptosis was carried out using a Muse analyzer. The results demonstrated a significant increase in the number of apoptotic cardiomyocytes in the 5-Fu group compared to the control group, whereas the combination group displayed a significantly reduced apoptosis rate compared to the 5-Fu group. Importantly, RES did not induce apoptosis in cardiomyocytes compared to the control group (Figure 5B and C).

The combined administration of RES and 5-Fu has demonstrated inhibitory effects on the growth of MFC cells through the regulation of p53-mediated autophagy and apoptosis. However, it remained unclear whether this effect would manifest in cardiomyocytes. Accordingly, western blot analysis was employed to assess the expression of apoptosis-related proteins and autophagy markers in cardiomyocytes. Surprisingly, the combination of RES and 5-Fu yielded contrasting results in cardiomyocytes. Specifically, when used individually or in combination, 5-Fu increased the expression of p53 compared to the control group, whereas RES had no effect on p53 expression in cardiomyocytes. Conversely, the co-administration of 5-Fu and RES led to a decrease in the expression of p53 protein, compared to the 5-Fu group (Figure 5D). Furthermore, the combined administration of 5-Fu and RES reversed the elevated levels of Bax observed in primary cardiomyocytes due to 5-Fu treatment. Notably, 5-Fu significantly inhibited the expression of Bcl-2 protein compared to the control group (P<0.01), whereas the combination of 5-Fu and RES resulted in an increase in Bcl-2 expression in cardiomyocytes (Figure 5E and F). In addition, the ratio of LC3-II/LC3-I in 5-Fu-treated cardiomyocytes was significantly higher than that in the control group. However, the combination of 5-Fu and RES reduced the LC3-II/LC3-I ratio compared to the 5-Fu group (Figure 5G-J). These findings suggested that RES can counteract the p53-mediated autophagy and apoptosis induced by 5-Fu in cardiomyocytes. These findings also suggested that RES possesses the potential to mitigate 5-Fu-induced cardiac damage and could serve as a promising therapeutic agent for the prevention or treatment of 5-Fu-induced cardiotoxicity.

## Discussion

Gastric cancer poses a significant global health challenge, and surgical intervention remains the primary treatment modality (5,22). Although multiple therapeutic options such as immunotherapy and targeted therapies are available for treating gastric cancer, 5-Fu-based chemotherapy remains one of the principal clinical approaches (23,24). However, 5-Fu administration can lead to both hepatotoxicity and cardiotoxicity, and effective clinical management strategies are currently lacking (25-27). The cardiotoxic effects of 5-Fu may manifest in several ways, including myocardial contractile dysfunction, arrhythmias, arterial vasospasm, direct endothelial cell toxicity, myocardial infarction (MI), heart failure (HF), and cardiogenic shock (28,29).

Presently, various clinical techniques are employed to prevent 5-Fu-induced cardiotoxicity (30). Eskilsson et al. proposed pre-administration of verapamil to mitigate 5-Fu cardiotoxicity due to vasospasm (31); however, the approach failed to alleviate symptoms. Another promising preventive measure involves the use of antioxidants such as probucol, which has demonstrated efficacy in laboratory settings, but its usefulness in human subjects is yet to be established (32). Thus, our study aimed to evaluate the potential cardioprotective effects of RES, a novel compound that may alleviate 5-Fu-induced cardiotoxicity.

According to recent research, RES exhibits antioxidant and anti-inflammatory properties. Moreover, it has been found that RES can inhibit the anti-cancer properties through changes in apoptotic signaling, metabolic pathways, and other signaling pathways that regulate key cellular processes including apoptosis, cell cycle progression, inflammation, proliferation, metastasis, and angiogenesis (33,34). Specifically, RES has been demonstrated to effectively induce apoptotic pathways, leading to the inhibition of cancer cell proliferation and an increase in susceptibility to drug resistance (35). In this study, the effects of RES on MFC cells were investigated, revealing a significant inhibition of proliferation and metastasis as well as an induction of apoptosis. Mechanistically, the observed effects of RES were attributed to the promotion of P53 protein expression and its synergistic effect with 5-Fu. Furthermore, it was observed that the expression of autophagy-related proteins LC3-I and LC3-II was increased in tumor cells treated with RES, indicating that RES could sensitize tumor cells to 5-Fu by activating apoptosis and autophagy pathways.

In the context of cardiovascular disease, RES has been reported to possess multiple beneficial effects. Specifically, it can ameliorate endothelial dysfunction by inhibiting endothelial cell proliferation and promoting endothelial nitric oxide production, resulting in vasodilation. RES can also mitigate oxidative stress and lipid oxidation, attenuate vascular inflammation, and inhibit platelet aggregation (36). These effects are achieved through the modulation of various molecular pathways, including cyclooxygenase 1, nuclear factor kappa B (NF-κB), deacetylase 1 (SIRT1), protein kinase B (PKB/Akt), nuclear factor-E2 related factor 2 (Nrf2), and AMP-activated protein kinase (AMPK), among others (37,38). Additionally, RES has demonstrated cardioprotective effects by reducing GPX4-dependent iron death induced by 5-Fu in H9C2 cells (39). 5-Fu has been demonstrated to adversely impact cardiac function through the elevation of oxidative stress in cardiomyocytes and the induction of apoptosis (40). Within our investigation, we observed that 5-Fu not only compromised cardiac function and resulted in altered cardiac morphology but also that RES effectively mitigated this effect. Additionally, RES not only reduced the level of oxidative stress in cardiomyocytes but also suppressed apoptosis and autophagy in these cells, indicating its potential to shield the heart from 5-FU-induced myocardial injury.

It is imperative to acknowledge that these findings are confined to animal experiments, and that further studies are essential to comprehensively elucidate the clinical potential of this combination therapy.

### Conclusion

In summary, the present study revealed that the co-administration of RES and 5-Fu can effectively impede the proliferation and metastasis of gastric cancer cells in a synergistic manner. Moreover, our results demonstrated that RES conferred protection against 5-Fu-induced cardiotoxicity by suppressing cardiomyocyte oxidative stress, apoptosis, and autophagy. Taken together, our findings highlight the potential of RES as a valuable adjunctive therapeutic agent to augment the efficacy of 5-Fu in the management of gastric cancer while mitigating the adverse effects on cardiac function.
